# Using a Machine Learning Approach to Monitor COVID-19 Vaccine Adverse Events (VAE) from Twitter Data

**DOI:** 10.3390/vaccines10010103

**Published:** 2022-01-11

**Authors:** Andrew T. Lian, Jingcheng Du, Lu Tang

**Affiliations:** 1 The Kinkaid School, Houston, TX 77024, USA; Andrew.Lian@kinkaid.org; 2 School of Biomedical Informatics, University of Texas Health Science Center at Houston, Houston, TX 77030, USA; Jingcheng.Du@uth.tmc.edu; 3 Department of Communication, Texas A&M University, College Station, TX 77843, USA

**Keywords:** COVID-19 vaccines, vaccine adverse events, Twitter, machine learning, natural language processing

## Abstract

Social media can be used to monitor the adverse effects of vaccines. The goal of this project is to develop a machine learning and natural language processing approach to identify COVID-19 vaccine adverse events (VAE) from Twitter data. Based on COVID-19 vaccine-related tweets (1 December 2020–1 August 2021), we built a machine learning-based pipeline to identify tweets containing personal experiences with COVID-19 vaccinations and to extract and normalize VAE-related entities, including dose(s); vaccine types (Pfizer, Moderna, and Johnson & Johnson); and symptom(s) from tweets. We further analyzed the extracted VAE data based on the location, time, and frequency. We found that the four most populous states (California, Texas, Florida, and New York) in the US witnessed the most VAE discussions on Twitter. The frequency of Twitter discussions of VAE coincided with the progress of the COVID-19 vaccinations. Sore to touch, fatigue, and headache are the three most common adverse effects of all three COVID-19 vaccines in the US. Our findings demonstrate the feasibility of using social media data to monitor VAEs. To the best of our knowledge, this is the first study to identify COVID-19 vaccine adverse event signals from social media. It can be an excellent supplement to the existing vaccine pharmacovigilance systems.

## 1. Introduction

As of December 2021, the COVID-19 pandemic has claimed over five million lives worldwide [1]. The COVID-19 vaccines have been proven to reduce infections, serious illness, hospitalizations, and dearth [2]. As new variants such as Delta and Omicron have emerged, the efficacy of the vaccines declined, but two shots and one booster still provide some protection again infection and solid protection against severe illness, hospitalization, and death [3,4]. As different COVID-19 vaccines have been distributed to billions of individuals worldwide, it is critical to continue to monitor the safety signals of vaccines and to track rare events. To that end, health authorities use both active surveillance such as Sentinel BEST (Biologics Effectiveness and Safety) and passive surveillance such as the Vaccine Adverse Event Reporting System (VAERS) to collect and share information about adverse events [5]. In addition to the traditional reporting channels established by governments and pharmaceutical companies such as VAERS, social media provides an opportunity for the surveillance of vaccine adverse events (VAEs), as social media users are likely to discuss their vaccination experiences on social media. 

Traditionally, social media data have been used for outbreak surveillance, i.e., using search records (such as Google Flu Trends, Mountain View, CA, USA) or the discussion of symptoms to track and predict the development of infectious disease outbreaks (for a systematic review, see Reference [6]). Flu surveillance is the oldest and most commonly used disease surveillance based on social media data mining [7]. More recently, social media data have been used to track other infectious disease outbreaks, such as Ebola [8], Zika virus [9,10], Dengue [11], and COVID-19 [12,13]. In addition, demographic information (such as age, gender, race, and geographic location) can allow researchers to identify those people who are at increased risk [14]. Several of these studies have calculated the temporal and spatial correlations between the outbreak magnitude shown on social media and the number of cases reported by the government and demonstrated the power of social media (such as Twitter, San Francisco, CA, USA and Sina Weibo, Beijing, China) contents in predicting the trajectory of different outbreaks (such as Zika, Dengue, and COVID-19 outbreaks) [10,11,12,13]. Social media outbreak surveillance is a cost-effective way to track the progression of infectious disease outbreaks and has been shown to detect hotspots of outbreaks before traditional outbreak surveillance conducted by public health agencies based on reports from physicians, clinics, and hospitals. Furthermore, social media-based disease surveillance could potentially contribute to a more accurate and comprehensive estimate of outbreaks, because it identifies disease cases even when individuals do not seek medical care from physicians and hospitals [15]. 

The same approaches can be leveraged in detecting and surveilling adverse events associated with medication, which is called pharmacovigilance [16]. According to the World Health Organization (the WHO), adverse drug events refer to any unexpected medical condition that appears due to the use of a pharmaceutical product [17]. Researchers have been using social media data for pharmacovigilance (for systematic reviews of this body of literature, see References [18,19]). Existing studies have utilized three primary social media sources: health care social networks and forums, general social networking sites, and search logs. Among these three, generic social networking sites (such as Twitter, Facebook, Menlo Park, CA, USA and Instagram, Menlo Park, CA, USA) allow for access to large and diverse populations across geographic areas but offer large volumes of data with high noise (i.e., data unrelated to the adverse events). In comparison, health-specific social media sites such as health forums and disease-specific forums provide more focused data about particular drugs but offer limited data [18]. Since more than 80% of adults in the US use the internet for health information and 72% use social media [20], search logs provide another tool for the identification of VAEs. In terms of methods, researchers have used an unsupervised lexicon-based approach or supervised classification based on annotated training datasets to identify the adverse effects of medication [19,21]. 

The ongoing COVID-19 pandemic necessitates the quick development, governmental approval, and rollout of different COVID-19 vaccines on a global scale. Today, over 8 billion doses of COVID-19 vaccines have been administered worldwide [1]. The WHO has given Emergency Use Listing (EUL) to seven COVID-19 vaccines (Pfizer/BioNTech (New York City, NY, USA), AstraZeneca (Cambridge, UK), Janssen (Johnson & Johnson) (New Brunswick, NJ, USA), Moderna (Cambridge, MA, USA), Sinopharm (Beijing, China), Sinovac (Beijing, China), and Bharat Biotech (Hyderabad, India)), with additional vaccines being administered in difficulty countries without WHO endorsement (such as the Sputnik V vaccine developed in Russia [22], Soberana, the Cuba’s homegrown COVID vaccine [23], and a variety of COVID-19 vaccines used in China [24]). As a result, it is of paramount importance for governments, medical establishments, and public health agencies to monitor the adverse events associated with these vaccines in a timely manner. While official reporting portals such as VAERS are essential, social media data can be used to provide additional complimentary information to these official sources. In addition, social media data can be even more important to the surveillance of vaccine adverse events where official data are not collected or unavailable. However, to our knowledge, there is no existing study using social media data to monitor vaccine adverse events in general and COVID-19 vaccines in particular. In this article, we proposed a supervised machine-learning-based system to identify COVID-19 vaccine adverse events signals from Twitter data and demonstrated the feasibility of this approach. 

## 2. Materials and Methods

**Figure 1:**
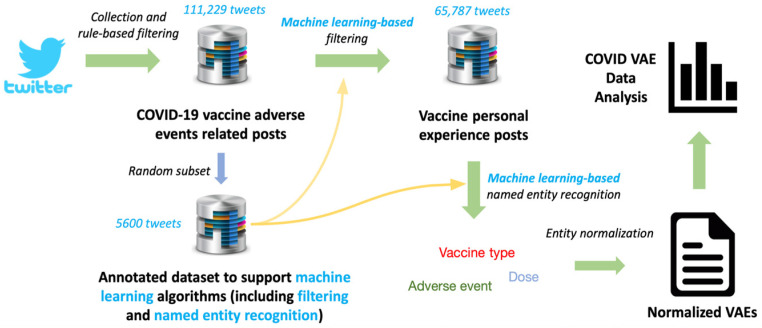
Method overview.

We collected COVID-19 vaccine-related discussions from Twitter using the Twitter streaming API. Tweets containing personal experiences postvaccination were identified (111,229) using a rule-based approach. We then randomly selected 5600 tweets for manual annotation. This annotated subset served as the training and test datasets for (1) machine-learning-based classification (i.e., further removing those tweets that are not personal experiences about vaccination) and (2) named entity recognition (i.e., extract mentions about vaccine type, dose, and symptom/adverse event). Next, we leveraged CLAMP, a state-of-the-art natural language processing (NLP) pipeline, for entity normalization [25]. Finally, we further analyzed the extracted VAE data based on the location, time, and frequency. The method overview can be seen in Figure 1. 

Data Collection and Rule-Based Filtering: We used the Twitter streaming API to collect vaccine-related discussions from December 2020 to August 2021 based on a set of keywords (e.g., Pfizer, Moderna, J&J, Johnson & Johnson, BioNTech, vaccine, AstraZeneca, covidvac, etc.). Since the Twitter data contained a high percentage of noise (tweets not related to COVID-19 vaccine adverse events), we then used rule-based approaches to remove the noise and identify the content related to personal experiences of COVID-19 vaccine adverse events. First, we removed retweets and quotes and only kept the original tweets so that the VAEs were not artificially inflated. Second, we removed the tweets from users with more than 10,000 followers, which are considered by Twitter as “super follows” [26], to focus on personal stories from ordinary users instead of organizations and popular influencers. Third, we selected only those tweets containing the word “vaccine” and at least one of the adverse event keywords. We selected the top 100 adverse events following COVID-19 vaccines from the VAERS databases. Then, we manually reviewed each one of the symptoms and added their synonyms as much as possible. Finally, we added a few of the symptoms that have drawn public attention recently, such as blood clots, thrombosis, etc. Finally, a keyword list containing 111 symptom names with variations was created. Finally, we selected tweets containing self-related keywords, such as i, my, mine, me, etc., to identify content related to personal experiences and exclude general discussions of VAEs. In the end, a total of 111,229 tweets remained after this filtering process. 

Machine Learning-Based Filtering: We built machine learning-based approaches to further select tweets that contained personal VAE experiences after COVID-19 vaccination. A set of commonly used machine learning algorithms, including support vector machine (SVM), logistic regression, random forest, extra trees, and gradient boosting, implemented using the scikit-learn package were evaluated [27]. 

Named Entity Recognition (NER): For tweets containing individual vaccine experiences, we further built a machine learning-based named the entity recognition (NER) model to extract information on (1) vaccine type (e.g., Pfizer, Moderna, or Johnson & Johnson); (2) dose (e.g., first dose or second dose); and (3) adverse event (e.g., fever, fatigue, or pain) from tweets. We leveraged CLAMP [25], an integrated clinical NLP toolkit, to implement a Conditional Random Fields (CRF) algorithm for this NER task. We also conducted normalization on the extracted entities (i.e., mapping the adverse events to the MedDRA Preferred Terms) [28]. 

**Figure 2:**
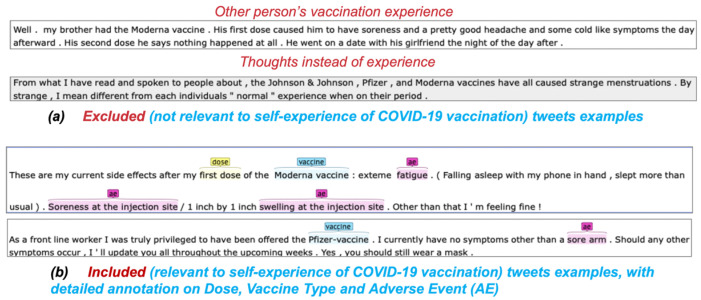
Process of annotation. (**a**) Two sample tweets that were excluded for further annotation (**b**) One sample tweet with annotation result.

Annotation and preprocessing: After rule-based filtering, we had 111,229 tweets that may contain COVID-19 VAE experiences. Three annotators coded 5600 tweets that were randomly selected to decide if a tweet contained personal VAE. The annotation agreement (measured as a F-1 score) was 0.96. We excluded 2216 tweets from this step, because they did not contain personal VAE. We further annotated the remaining 3384 tweets in terms of vaccine type, dose, and adverse events. Cohen’s kappa was calculated to measure the intercoder reliability. The agreement scores were 0.83, 0.84, and 0.82, respectively, which indicated strong agreement [29]. Figure 2 shows the process of annotation.

Evaluation: The annotated tweets were divided into training, validation, and testing sets with the proportion of 7:1:2 for both the text classification tasks and named entity recognition tasks. We calculated the precision, recall, and F-1 scores for the two tasks. These three measures are commonly used in evaluating the performance of machine learning algorithms. Precision calculates the percentage of correct positive predictions out of all the positive predictions (true positive/(true positive + false positive)). Recall is the percentage of positive prediction from all the positive cases in the data (true positive/(true positive + false negative)). The F-1 score is the harmonic mean of precision and recall, where F-1 is 2*precision*recall/(precision + recall).

Table 1 shows a comparison of the results of the selected machine learning algorithms for tweet classification. Overall, these algorithms all performed quite well in this task. Among them, random forest (RF) achieved the best F-1 score (0.926) for the identification of VAE tweets. We further applied the trained RF classifier to other unlabeled tweets. In total, 65,787 tweets were selected by ML as containing personal VAE experiences. 

Named Entity Recognition and Normalization: For the tweets containing personal vaccine experiences included in this study, we leveraged a trained CRF algorithm and a few lexicon-based rules through CLAMP. Table 2 shows the precision, recall, and F-1 scores for our algorithm to extract each type of the entities. Overall, our algorithm achieved a relatively good performance, with F-1 scores ranging from 0.770 to 0.857 across three tasks. We then applied the algorithm and extracted 66,499, 27,709, and 69,177 mentions of adverse events, doses, and vaccine types, respectively. 

## 3. Results

**Figure 3:**
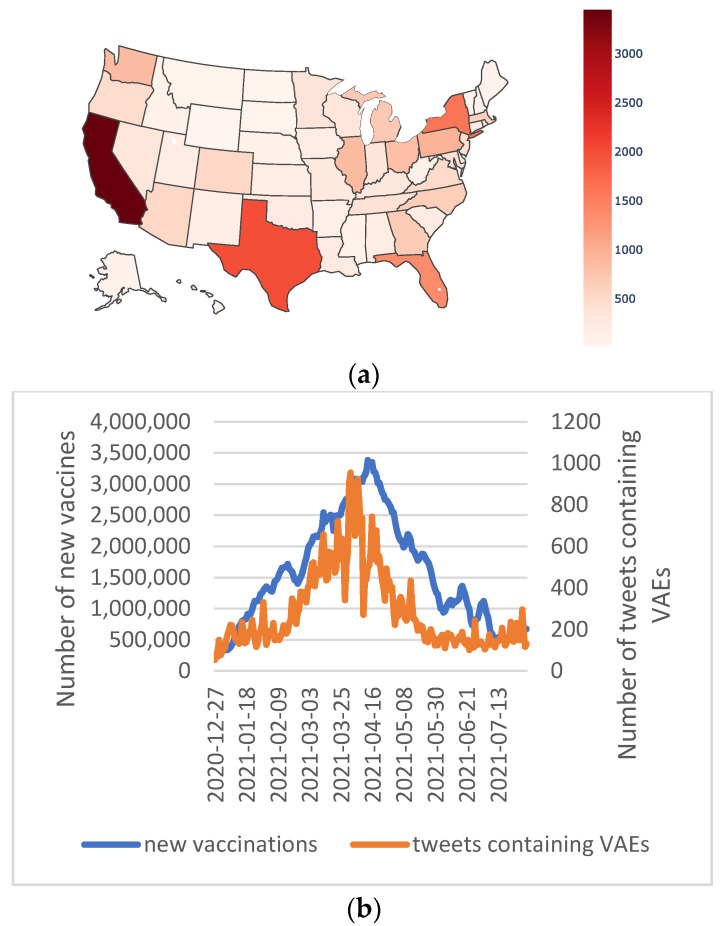
(**a**) Distribution of tweets containing self-experienced COVID-19 VAEs by US states. (**b**) VAE tweet temporal trends and new vaccination numbers in the United States (new vaccination numbers from Ritchie 2021).

We extracted the geographic locations of these tweets and analyzed the longitudinal trends of the VAE-related tweet volumes. Even though our data included 66,499 VAE mentions, only around 30% of them (*n* = 23,657) included geographic information. Figure 3a shows the distribution of tweets containing COVID-19 VAEs in different states in the US. The top four states that tweeted the most about COVID-19 vaccine adverse effects were also the four most populated states in the United States: California, Texas, Florida, and New York and had the doses of COVID-19 vaccines administered [1]. It was natural that the states with the most vaccinations were also the states with the highest numbers of VAEs reported on social media. 

Figure 3b shows the temporal trends of personal VAE discussions (red line), as well as the longitudinal changes in the new vaccinations each day (blue line, data from Reference [1]) over the same period of time. These two lines showed consistent longitudinal trends, and both the number of new vaccinations and the number of tweets containing VAEs peaked in April 2021.

**Figure 4:**
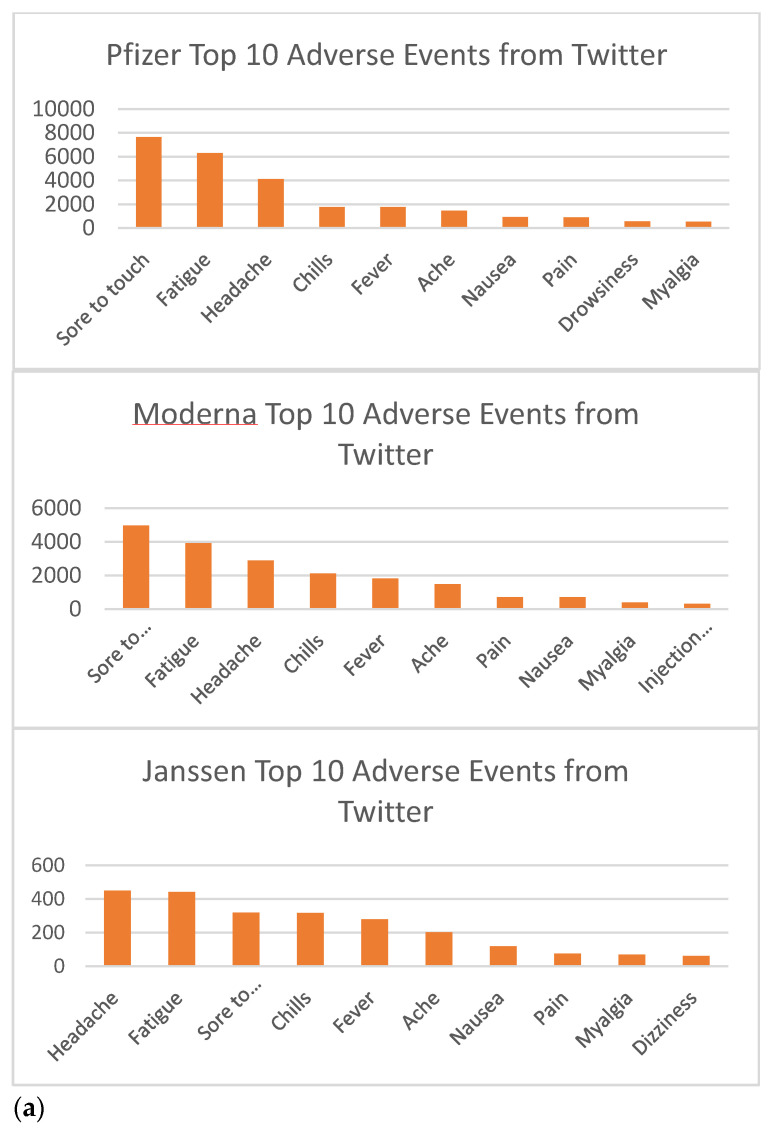

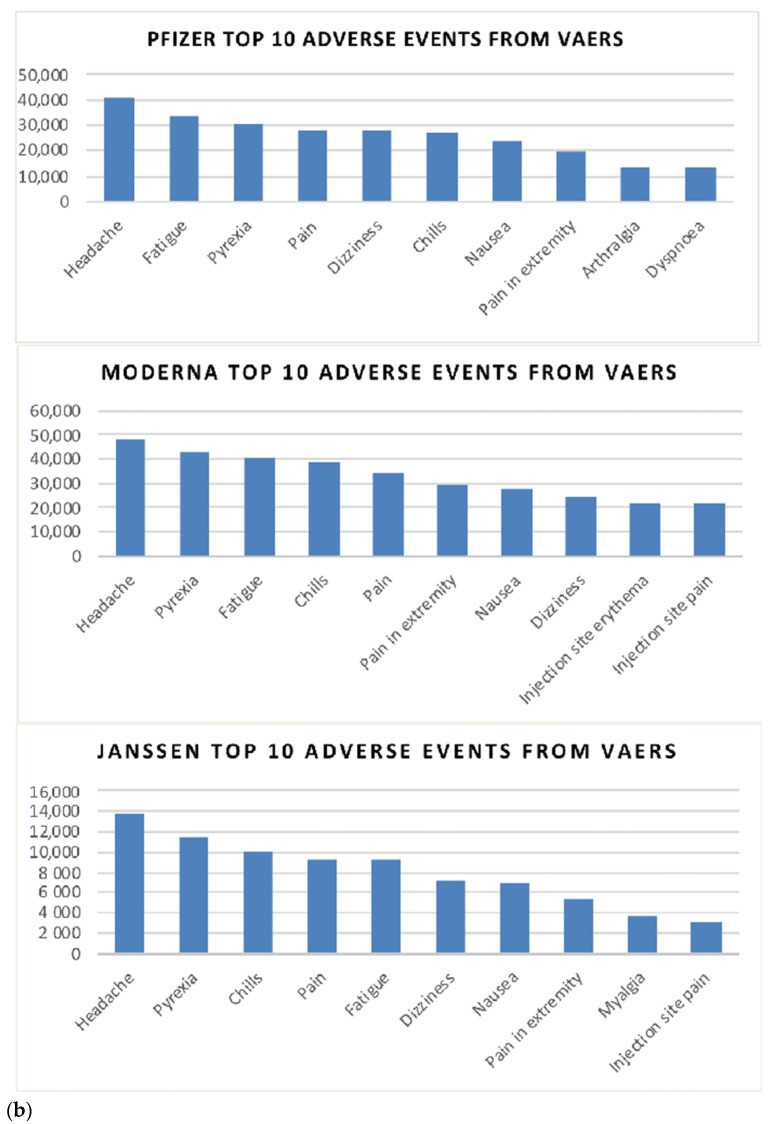
(**a**) The most frequently mentioned VAEs by Twitter users. (**b**) The most frequently mentioned VAEs in VAERS.

We further calculated the top 10 most frequently discussed adverse events for each of the three COVID-19 vaccines available in the United States (see Figure 4a). Sore to touch, fatigue, and headaches were the top three most common adverse events for all three vaccines. All these top events appeared to be mild symptoms. We also tabulated the top 10 adverse events of these three vaccines reported in the Vaccine Adverse Event Reporting System (VAERS) operated by the US government’s Department of Health and Human Services (See Figure 4b). A comparison between the results of our Twitter-based results and the VAERS data yielded some differences. For instance, “sore to touch” was the most frequent VAE for the Pfizer and Moderna vaccines and the third-most frequent VAE for the J&J vaccine, but it was not listed in the VAERS data. Other than that, we observed consistency between the VAEs identified through Twitter and found in VAERS based on medical reports. 

## 4. Discussion

According to the Pew Research Center, seven out of ten Americans used social media in 2021 [30]. The public often shares their healthcare experiences, including their experiences with vaccines, on social media. Such contents can be leveraged for the monitoring of vaccine adverse events, as well as individual attitudes and behaviors related to disease outbreaks and vaccines [31]. In this paper, we proposed a machine-learning-based approach to identify the VAEs related to COVID-19 vaccines based on Twitter data. To the best of our knowledge, this study represents the first effort to identify COVID-19 vaccine adverse events signals from social media. Compared to the surveillance of adverse events other drugs that are only used by a small percentage of the population, COVID-19 vaccine adverse events are especially well-aligned with the analysis of social media data, because over 70% of the US population has received at least one dose of a COVID-19 vaccine [32]. 

Using both machine learning and named entity recognition, our approach can relatively accurately identify the vaccine type, dose, and vaccine adverse events from COVID-19 vaccine-related tweets based on a small training dataset. A comparison of the SVM, extra trees, random forest, logistic regression, and gradient boosting algorithms showed that random forest has the best performance. Our results showed that the four most populous states in the US also had the most discussions of VAEs on Twitter. The trends of VAE-related tweets are also consistent with the US vaccination progress, which further demonstrates the validity of our machine learning-based pipeline.

In comparing our results to the data reported in the Vaccine Adverse Event Reporting System, we found that “sore to touch” was the most common VAE identified through Twitter data but did not show up in the VAERS. This is probably because “sore to touch” is such a minor yet common adverse event that individuals do not bother to report it to physicians and through the governmental portal, but they nevertheless talk about it in describing their vaccine experiences on Twitter. This discrepancy shows the relative strength and weakness of identifying VAEs through social media content that people share informally and through more formal report portals operated by the government and pharmaceutical companies. 

Using Twitter data to monitor VAEs is not without its limitations. Twitter has been the most often used social media platform for outbreak surveillance and adverse effect surveillance in recent years [6] due to the public nature of the data. However, generalizability is a problem that researchers need to keep in mind. For instance, although the VAEs identified through Twitter data were typically mild side effects, it is conceivable that those people suffering from severe VAEs are less likely to discuss their experiences on Twitter than those experiencing mild VAEs. Furthermore, due to the demographic profile of Twitter users [33], such data mining-based surveillance realistically only monitors a younger part of the population. Those parts of the population that do not have access to the internet or social media are left out (such as senior citizens or individuals of lower socioeconomic status or those live in remote areas without broadband internet). This poses the question of equity [15]. Language is another bias, as our approach only captured English language tweets. However, the method developed in this study can potentially be used to monitor VAEs in non-English tweets or contents on other social media platforms. In addition, even though public health surveillance based on social media data has been ongoing for more than a decade, the research community is still grappling with the issues of privacy and availability of social media contents, which are technically the property of companies [15].

Furthermore, research has shown that a fear of vaccine adverse effects contributes to vaccine hesitancy and called for a “positive framing of mild adverse effects” and “balancing risk and benefit information” [34]. Understanding how people talk about VAEs on social media can provide public health agencies and physicians with the base data to communicate to the public and patients. While this study only used Twitter data to identify the discussion of VAEs, other studies have mined vaccine-related sentiments, attitudes, and information from Twitter data [35]. In the future, these different types of data could be integrated to provide a more comprehensive picture of the public opinion, experiences, and sentiments about vaccines, as well as other health-related issues. For instance, VAEs can be discussed using the gain frame, emphasizing the benefits of vaccines, or using the loss frame, focusing on the harm of not getting vaccines. Past research has shown that such frames can influence an audience’s responses to vaccine-related messages [36]. Alternatively, the VAE information could be analyzed in conjunction with the emotions expressed in these tweets, because past research has shown that negative emotions contribute to vaccine hesitancy and positive emotions lead to vaccine confidence [37].

## 5. Conclusions

The ongoing COVID-19 pandemic is the most significant threat to global health, and vaccines are one of the most effective tools in our collective fight against COVID-19. Vaccine safety is of paramount importance, and the perceived lack of safety is a major barrier to vaccine rollout. In addition to/instead of reporting their advertise events to their physicians and the portals created by the government and pharmaceutical companies, individuals often share their vaccine experiences and adverse events on social media. Our proposed system could be a good supplement to existing vaccine pharmacovigilance systems.

## Figures and Tables

**Table 1:** Comparison of ML algorithms for tweet classification, implemented using the scikit-learn package.

Algorithm		SVM	Extra Trees	Random Forest	Logistic Regression	Gradient Boosting
Precision	Included	0.896	0.904	0.906	0.927 *	0.921
	Excluded	0.898	0.852	0.910 *	0.878	0.850
Recall	Included	0.940	0.905	0.946 *	0.921	0.901
	Excluded	0.830	0.850	0.847	0.886 *	0.879
F-1	Included	0.918	0.905	0.926 *	0.924	0.911
	Excluded	0.863	0.850	0.877	0.882 *	0.864
Accuracy		0.897	0.884	0.908 *	0.908 *	0.892

* Represents the best performing algorithm for different performance metrics.

**Table 2:** Performance of the named entity recognition through the Conditional Random Fields (CRF) algorithm provided by CLAMP.

Entity	Vaccine	Dose	Adverse Event
Precision	0.856	0.851	0.784
Recall	0.846	0.862	0.755
F-1	0.851	0.857	0.770

## Data Availability

The data that support the findings of this study are available from the corresponding author upon request. The data are not publicly available due to privacy concerns for Twitter users.

## References

[1] Ritchie H., Mathieu E., Rodés-Guirao L., Appel C., Giattino C., Ortiz-Ospina E., Hasell J., Macdonald B., Beltekian D., Dattani S. (2021). Coronavirus Pandemic (COVID-19). Our World in Data. https://ourworldindata.org/coronavirus.

[2] CDC (2021). CDC Ensuring COVID-19 Vaccines Work. https://www.cdc.gov/coronavirus/2019-ncov/vaccines/effectiveness/work.html.

[3] Reis B.Y., Barda N., Leshchinsky M., Kepten E., Hernan M.A., Lipsitch M., Daga N., Balicer R.D. (2021). Effectiveness of BNT162b2 Vaccine against Delta Variant in Adolescents. N. Engl. J. Med..

[4] Collie S., Champion J., Moultrie H., Bekker L.-G., Gray G. (2021). Effectiveness of BNT162b2 Vaccine against Omicron Variant in South Africa. N. Engl. J. Med..

[5] U.S. Food & Drug Administration (2021). COVID-19 Vaccine Safety Surveillance. https://www.fda.gov/vaccines-blood-biologics/safety-availability-biologics/covid-19-vaccine-safety-surveillance.

[6] Gupta A., Katarya R. (2020). Social media based surveillance systems for healthcare using machine learning: A systematic review. J. Biomed. Inform..

[7] Eysenbach G. (2006). Infodemiology: Tracking flu-related searches on the web for syndromic surveillance. AMIA Annu. Symp. Proc..

[8] Hossain L., Kam D., Kong F., Wigand R.T., Bossomaier T. (2016). Social media in Ebola outbreak. Epidemiol. Infect..

[9] Abouzahra M., Tan J. (2021). Twitter vs. Zika—The role of social media in epidemic outbreaks surveillance. Health Policy Technol..

[10] Masri S., Jia J., Li C., Zhou G., Lee M.-C., Yan G., Wu J. (2019). Use of Twitter data to improve Zika virus surveillance in the United States during the 2016 epi-demic. BMC Public Health.

[11] Carlos M.A., Nogueira M., Machado R.J. Analysis of dengue outbreaks using big data analytics and social networks. Proceedings of the 2017 4th International Conference on Systems and Informatics (ICSAI).

[12] Li J., Xu Q., Cuomo R., Purushothaman V., Mackey T. (2020). Data mining and content analysis of the Chinese social media plat-form Weibo during the early COVID-19 outbreak: Retrospective observational infoveillance study. JMIR Public Health Sur-Veill..

[13] Shen C., Chen A., Luo C., Zhang J., Feng B., Liao W. (2020). Using Reports of Symptoms and Diagnoses on Social Media to Predict COVID-19 Case Counts in Mainland China: Observational Infoveillance Study. J. Med. Internet Res..

[14] Chen J., Wang Y. (2021). Social Media Use for Health Purposes: Systematic Review. J. Med. Internet Res..

[15] Aiello A.E., Renson A., Zivich P.N. (2020). Social Media– and Internet-Based Disease Surveillance for Public Health. Annu. Rev. Public Health.

[16] Nikfarjam A., Gonzalez G.H. (2011). Pattern mining for extraction of mentions of adverse drug reactions from user comments. AMIA Annu. Symp. Proc..

[17] World Health Organisation (2002). Safety of Medicines—A Guide to Detecting and Reporting Adverse Drug Reactions-Why Health Professionals Need to Take Action.

[18] Pappa D., Stergioula L.K. (2019). Harnessing social media data for pharmacovigilance: A review of current state of the art, challenges and future directions. Int. J. Data Sci. Anal..

[19] Tricco A.C., Zarin W., Lillie E., Jeblee S., Warren R., Khan P.A., Robson R., Pham B., Hirst G., Straus S.E. (2018). Utility of social media and crowd-intelligence data for pharmacovigilance: A scoping review. BMC Med. Inform. Decis. Mak..

[20] Chou W.-Y.S., Gaysynsky A., Trivedi H., Vanderpool R.C. (2021). Using Social Media for Health: National Data from HINTS 2019. J. Health Commun..

[21] Paul M.M., Sarker A., Brownstein J.S., Nikfarjam A., Scotch M., Smith K.L., Gonzalez G. (2016). Social media mining for public health monitoring and surveillance. Pacific Symposium on Biocomputing 2016.

[22] Jones I., Roy P. (2021). Sputnik V COVID-19 vaccine candidate appears safe and effective. Lancet.

[23] Taylor L. (2021). Why Cuba developed its own covid vaccine—And what happened next. BMJ.

[24] World Health Organisation (2021). Coronavirus Disease (COVID-19): Vaccines. https://www.who.int/news-room/questions-and-answers/item/coronavirus-disease-(covid-19)-vaccines?gclid=CjwKCAiAh_GNBhAHEiwAjOh3ZJ1mu-mpXy9QnPivLAUOmBwM9c8PRANGjjtHJydxTOWdzQQQUTPs4RoCEqEQAvD_BwE&topicsurvey=v8kj13.

[25] Soysal E., Wang J., Jiang M., Wu Y., Pakhomov S., Liu H., Xu H. (2018). CLAMP—A toolkit for efficiently building customized clinical natural language processing pipelines. J. Am. Med. Inform. Assoc..

[26] Crawford E. (2021). Introducing Super Follows. https://blog.twitter.com/en_us/topics/product/2021/introducing-super-follows.

[27] Pedregosa F., Varoquaux G., Gramfort A., Michel V., Thirion B., Grisel O., Blondel M., Prettenhofer P., Weiss R., Dubourg V. (2011). Scikit-learn: Machine Learning in Python. J. Mach. Learn. Res..

[28] Medical Dictionary for Regulatory Activities. https://www.meddra.org/.

[29] McHugh M.L. (2012). Interrater reliability: The kappa statistic. Biochem. Med..

[30] Auxier B., Anderson M. (2021). Social Media Use in 2021.

[31] Tang L., Bie B., Park E.S., Zhi D. (2018). Social media and outbreaks of emerging infectious diseases: A systematic review of literature. Am. J. Infect. Control.

[32] Mayo Clinic U.S. COVID-19 Vaccine Tracker: See Your State’s Progress. https://www.mayoclinic.org/coronavirus-covid-19/vaccine-tracker.

[33] Wojcik S., Hughes A. (2019). Sizing Up Twitter Users.

[34] Rief W. (2021). Fear of adverse effects and COVID-19 vaccine hesitancy: Recommendations of the treatment expectation expert group. JAMA Health Forum.

[35] Du J., Tang L., Xiang Y., Zhi D., Xu J., Song H., Tao C. (2018). Public Perception Analysis of Tweets During the 2015 Measles Outbreak: Comparative Study Using Convolutional Neural Network Models. J. Med. Internet Res..

[36] Penţa M.A., Băban A. (2018). Message Framing in Vaccine Communication: A Systematic Review of Published Literature. Health Commun..

[37] Chou W.-Y.S., Budenz A. (2020). Considering Emotion in COVID-19 Vaccine Communication: Addressing Vaccine Hesitancy and Fostering Vaccine Confidence. Health Commun..

